# F121. DOES RELAPSE CONTRIBUTE TO TREATMENT RESISTANCE? ANTIPSYCHOTIC RESPONSE IN FIRST- VS. SECOND-EPISODE SCHIZOPHRENIA

**DOI:** 10.1093/schbul/sby017.652

**Published:** 2018-04-01

**Authors:** Hiroyoshi Takeuchi, Cynthia Siu, Gary Remington, Gagan Fervaha, George Foussias, Robert Zipursky, Ofer Agid

**Affiliations:** 1 ​Keio University School of Medicine; 2 COS and Associates Ltd; 3 Centre for Addiction and Mental Health (CAMH), University of Toronto; 4 Queen’s University Faculty of Medicine; 5 McMaster University

## Abstract

**Background:**

The objective of this study was to compare trajectories of antipsychotic response before and after relapse following response from a first episode of schizophrenia or schizoaffective disorder.

**Methods:**

The current analysis included patients with a diagnosis of first-episode schizophrenia or schizoaffective disorder who met the following criteria: (1) referral to the First-Episode Psychosis Program between 2003 and 2013; (2) treatment with an oral second-generation antipsychotic according to a standardized treatment algorithm; (3) positive symptom remission; (4) subsequent relapse (i.e., second episode) in association with non-adherence; and (4) reintroduction of antipsychotic treatment. The following outcomes were used as an index of antipsychotic treatment response: change in the Brief Psychiatric Rating Scale (BPRS) total score and number of patients who achieved positive symptom remission, including 20% and 50% response improvement.

**Results:**

A total of 130 patients were included in the analyses. All patients took the same antipsychotic in both episodes. Antipsychotic doses in the second episode were significantly higher than those in the first episode (P=0.03). There were significant episode-by-time interactions for all outcomes of antipsychotic treatment response over 1 year (all Ps<0.001) in favor of the first episode compared to the second episode. Results remained unchanged after adjusting for antipsychotic dose.

**Discussion:**

The present findings suggest that antipsychotic treatment response is reduced or delayed in the face of relapse following effective treatment of the first episode of schizophrenia.

